# Navigating complexity: key considerations for studying fungal-bacterial interactions

**DOI:** 10.1128/msystems.01728-25

**Published:** 2026-04-21

**Authors:** Gaëtan Martin, Giovana S. Slanzon, Ishwora Dhungana, Rishi R. Prasadh, Caio César Pires de Paula, Nhu H. Nguyen

**Affiliations:** 1 Department of Tropical Plant and Soil Sciences, University of Hawai‘i at Mānoa, Honolulu, Hawaii, USA; 2 Department of Ecosystem Biology, Faculty of Science, University of South Bohemia, České Budĕjovice, Czech Republic; 3 Institute of Hydrobiology, Biology Centre CAS, České Budĕjovice, Czech Republic; Tufts University, Medford, Massachusetts, USA

**Keywords:** interactions, bacterial-fungal interactions, BFI, microbial theory, cross-kingdom, cross-domain, interaction phases

## Abstract

Fungal-bacterial interactions are widespread phenomena that are currently gaining attention across diverse research fields. But what are interactions, how do we identify them, and why should we adopt ecological theory when studying them? While microbial interactions are often conceptualized and characterized as static properties, these relationships are dynamic and complex. They are shaped by the interplay of numerous variables, which themselves fluctuate over space and time, that scale from molecules to ecosystems. In this perspective, we discuss the theoretical and practical challenges of studying the interactions between these two ubiquitous and diverse groups of microorganisms and propose a framework grounded on mechanistic and systems approaches rather than relying on correlations or fragmented practices. We hope to inspire efforts to build and integrate a more comprehensive understanding of this fascinating and quickly growing subdiscipline of microbial ecology.

## INTRODUCTION

The interactions between fungi and bacteria, two of the most taxonomically and metabolically diverse groups of microorganisms, exist in a system of complexity that requires theoretical approaches to disentangle. Generally, they share similar niches across diverse ecosystems, resulting in dynamic multipartite interactions that scale both space and time (1–4). As such, these complex interactions are an inherent property of the microbial world. However, understanding the nature of fungal-bacterial interactions (or bacterial-fungal interactions) comes with specific challenges as each group of interacting partners has its own physiology, distinct morphology, and life history. (The ordering of the term “fungal-bacterial interactions” used in this paper as opposed to the more common “bacterial-fungal interactions” [BFI] is more than just a preference. We think of fungi as both a host and a symbiont, where large portions of their tissues [mycelium or mushrooms, or individual hyphae when associating with endohyphal bacteria] serve as the host, whereas individual hyphae are symbionts that interact at the same level as their bacterial counterparts. Therefore, the ordering of the term fungal-bacterial interactions is a deliberate emphasis within the host-symbiont framework, acknowledging that fungi bridge both the microbial and macrobial world.) Because fungi and bacteria operate at different spatial and temporal scales, their interactions act as bridges across those scales, leading to features such as hierarchical structure, feedback loops, history dependence, and self-organization (5, 6). These are characteristics of complex systems, which in turn implies that their behavior is hard to predict due to non-linearity, chaotic behavior, and emerging properties (7).

While the application of ecological theory to microbial ecology is well established, fungal-bacterial interactions present an underutilized system for theory testing. Their unique characteristics, such as the coexistence of organisms with distinct spatial strategies, metabolic capacities, and growth dynamics, make them ideal for testing fundamental questions in coexistence theory, niche construction, or spatial self-organization (8). Yet, the significant challenges of studying fungal-bacterial interactions come with potentially high rewards, including improved understanding of complex ecological systems while leading breakthroughs in fields critical to our societies with potential impact on our health (9), food and energy production (10), and the preservation of our environment (11).

**Fig 1:**
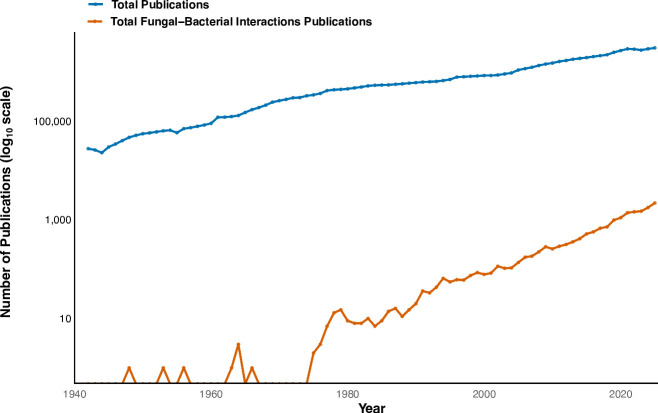
Increasing trend of fungal-bacterial interaction papers relative to total publications across all fields since 1940. From 1980 to 2025, the number of publications that match the search terms representing fungal-bacterial interactions rose two orders of magnitude, from 10 papers to 2,100 per year. While the trend since the 1980s reflected the overall trend in total publications, the topic of fungal-bacterial interactions remains two orders of magnitude below the total publication rate. This suggests that the field is growing strongly, with the publication rate tracking the growing trend in publications over time. Publication data were retrieved from the Web of Science Core Collection using the Advanced Search interface. The total number of indexed publications was obtained using the query DT=(Article OR Review) AND PY=1942–2025. All document types were included. Publications related to fungal-bacterial interactions were identified using the field search (bacteri* AND fung* AND interaction*). For both searches, annual publication frequencies were extracted using the “Analyze Results” tool under the “Publication Years” category. Records associated with fungal-bacterial interactions represent a subset of the total publications and are therefore included within the total publication counts.

Until more recently, mycology and bacteriology have existed as distinct research fields without theoretical (or even conceptual) integration of how these organisms might interact with each other. For example, despite one of the most important scientific advances of the 20th century—the groundbreaking discovery of the antibacterial properties of *Penicillium*—microbial ecologists have long overlooked the ecological significance of fungal-bacterial interactions. Almost a century after Alexander Fleming’s discovery, the interest in elucidating the mechanisms and outcomes of fungal-bacterial interactions is rising. During the last few decades, the number of published studies in Web of Science for fungal-bacterial interactions jumped from less than 10 papers in 1980 to more than 2,100 in 2024 (Fig. 1). This increase in the proportion of fungal-bacterial interaction papers relative to overall microbial interaction papers, the multiple review papers on the topic (6, 9, 10, 12–19), and the many recent conference proceedings indicate that this field is gaining momentum. We are particularly excited by this trend, but we also found that with many new and emerging fields, there is no grounding theoretical framework to connect the vastly complex, intricate ecosystems, characterized by multifaceted interactions that scale across various levels. Specifically, we lack a solid framework to study fungal-bacterial interactions.

Given the growing interest in the topic, this is an opportune moment to initiate dialogue with the rapidly growing research community and build a general theoretical framework to study fungal-bacterial interactions. Assessing how general ecological theory applies to microbial ecology, especially across domains (“cross-kingdom”), is essential for building a unified synthesis of ecology across all life forms (20). Ecologists working across major systems have developed and tested numerous theoretical approaches that might be adapted for fungal-bacterial interactions. Therefore, we propose that this fungal-bacterial interactions framework must incorporate the idea of complexity and complex systems, backed by ecological theory (7). Thoughtfully built, this framework will allow us to move away from producing a mere collection of loosely tied case studies to creating actionable research that not only advances understanding but also informs decisions, predictions, and interventions with societal relevance (20).

Although studying fungal-bacterial interactions comes with many challenges, both practical and conceptual, we are optimistic that as a community, we can collectively tackle those issues to develop a robust framework with actionable knowledge. Here, we will discuss key concepts critical to untangling the complexity of fungal-bacterial interactions (see Table 1 for definitions that guide these concepts). We focus on theoretical approaches and not on the technical methods, which have been reviewed elsewhere (6, 21, 22). We hope that this will stimulate discussion among veterans of the field and serve as a gateway to explore key concepts for those just entering the field. When thoughtfully considered and carefully implemented, theory-based fungal-bacterial interaction research should provide a concerted movement of the field forward.

**TABLE 1:** Definition of scales and features of complex systems used in this manuscript, adapted from Riva et al. (7)

Concept	Definition	Class
Community scale	The scope at which ecological interactions are considered within a system. It can be described by the number of organisms participating in the system, affecting the system, or being affected by the system	Scale
Spatial scale	The physical extent at which interactions unfold includes the dwelling scales of fungi and bacteria. These ranges may encompass different environments, including physical links among multiple environments	Scale
Time scale	The temporal dimension of interactions, including the characteristic events and their duration, that shape system dynamics (e.g., steady states, perturbations, life history traits, circadian rhythms, or Lotka–Volterra cycles). It also covers the four phases of an interaction proposed here	Scale
Attractor	One of several configurations a system tends to move toward	System behavior
Context dependency	The same components or processes can produce different effects under different conditions	System behavior
Scaling	The tendency of system property to change with spatial, temporal, or taxonomic scale	System behavior
Threshold	The point at which a small change in system conditions produces a disproportionately large change in the system itself. This concept is equivalent to the “single pulse hypothesis” *sensu* Barrios (23)	System behavior
Feedback loop	A recursive process through which the consequences of a system’s actions influence its future functioning, either amplifying or counteracting change	Structural property
Network structures	Interconnected sets of components (nodes and edges) displaying non-trivial topological features (i.e., clustering or heterogeneous distribution of connections)	Structural property
Self-organization	The emergence of dynamics, organized structures, or system-level patterns driven by local interactions	Structural property
Adaptation	System modification in response to changes in external or internal factors or states	Functional property
Chaos	A deterministic yet unpredictable system, where tiny changes in initial parameters can yield vastly different outcomes	Functional property
Homeostasis	Self-regulating mechanisms through which a system maintains its internal balance and continued operation	Functional property
Resilience	The ability of a system to recover its stability after disturbance	Functional property
Stability	The ability of a system to maintain equilibrium by resisting perturbation	Functional property

## WHAT ARE INTERACTIONS?

Characterizing interactions among organisms is central to ecology, but the definition of interaction is broad and inexact, often only contextualized through analogies. These range from socio-economic markets resembling how microorganisms cooperatively or competitively “trade” goods or services (24), social networks modeling how relationships can mutually impact behavior (25, 26), game theory where microbes play strategies aiming to maximize their survival and fitness (27), and hierarchical organizations, in which specific microbes are responsible for specific roles within a community (28). However, it is surprisingly difficult to find an operational definition of interaction in ecology, even in articles that aim to teach ecological interactions (29). Typically, the literature focuses on identifying the type of interactions (e.g., positive/mutualism/facilitation, neutral/commensalism/amensalism, negative/antagonism/competition) that underlie ecological principles, such as competitive exclusion and trophic cascade, but assumes that the definition of interactions is of common knowledge. Recently, Meroz et al. (30) defined biological interactions as the “mutual influences between individuals, where the presence or actions of one individual affect those of another.” We propose that this definition should be expanded to include that of Bahr and Stary (31), where interactions should also consider “the exchange of material or immaterial goods between acting parties (biological or technical entities) embodied in a certain context.” This addition includes situations where organisms might exchange resources with no detectable influence (e.g., genotypic, phenotypic, or behavioral traits) on each other, influences that may be asymmetric and not mutual, or those not detected at specific time points (23). Such interactions might be dismissed under the first definition but would be of importance in a highly connected and time-dependent system (32).

**Fig 2:**
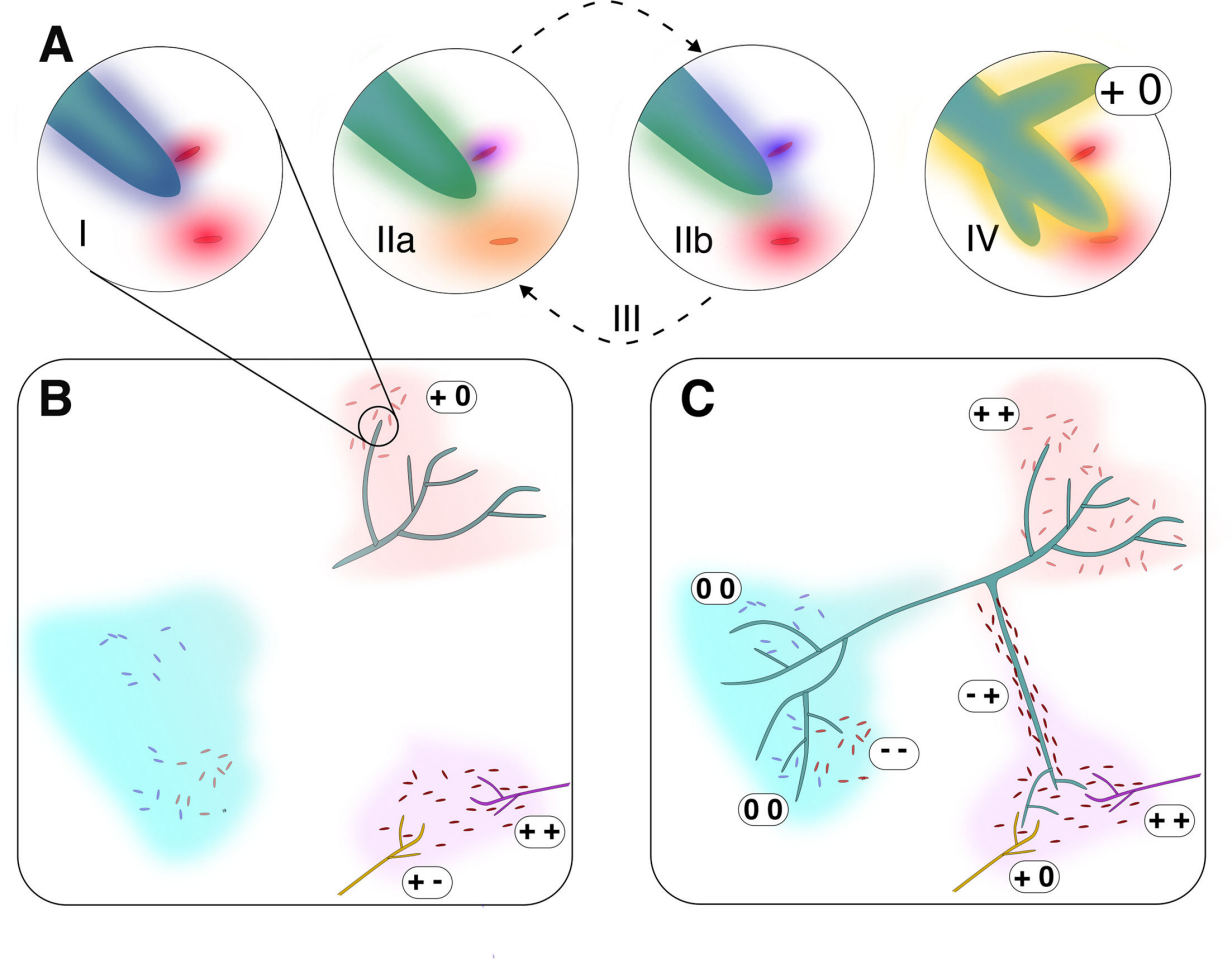
The system properties of fungal-bacterial interactions across time and space. (**A**) Transition among the four phases of interactions. Interaction begins when the organisms detect one another through chemical cues (purple and red halos) or physical contact (**I**), triggering one or multiple physiological responses in one or both partners (IIa and IIb). These physiological shifts (represented by different colored halos) can influence the trajectory of the interaction through feedback mechanisms (III). The reciprocal exchanges will continue to occur until the system reaches a stable state (IV). The outcome of the interactions may be classified as positive, neutral, or negative from the perspective of each organism (39). In this example, the outcome is positive for the fungus and neutral for the bacteria (+ 0). (**B and C**) Multiple subsystems may exist with their own interacting partners, separated both spatially and temporally. As the partners expand across spatial and temporal scales, they transition from simple pairwise associations (**B**) into progressively more complex configurations that incorporate additional populations and communities (**C**). Over time, this expansion gives rise to a complex system consisting of a broader network of interacting subsystems. Note that this figure only notates the outcomes of pairwise interactions and not higher-order outcomes, such as alteration of the structure, function, or (alternative) stable states of the entire system. In natural settings, interactions with multiple partners, including indirect interactions with one of the partner pairs (e.g., an antagonist of a bacterium negatively interacting with a fungus, or phages and mycoviruses that shift interaction dynamics), are likely to occur (40, 41). + indicates positive interactions, (0) indicates neutral interactions, and – indicates negative interactions.

However, a complete definition of interactions relevant to fungal-bacterial interactions necessitates the placement of influences and measurable exchanges into a time-dependent, scalable, and experimentally tractable framework, which we propose here as the four interconnected phases of interactions: sense, response, feedback, and outcome (Fig. 2). The initiation of interaction (I) is to sense the presence of another entity, which typically occurs through detection of signal molecules (33, 34), whether they are soluble, insoluble, or volatile (35). Sensing of the molecule(s) may then elicit a response (II) that typically starts with transcriptional regulation, as observed in the case of the well-described two-component signaling systems present in both bacteria and fungi (36). The interacting partner(s) will elicit its own response that can result in feedback (III) (37, 38). The reciprocal exchanges will continue to occur until the system either (re)equilibrates (i.e., dynamics will reach a stable state where the overall ecological roles are fulfilled and maintained) or will collapse, suffering a catastrophic disturbance that drastically alters the structure and function of the system, resulting in an alternative stable state (IV). Both of these scenarios represent a potential outcome of the interaction or a system of interactions. These four phases are experimentally tractable, each with numerous methods available that allow us to describe, measure, and quantify them. They are scalable, whether measured as individual cells or as communities of organisms. And as they are inherently connected through time, linking them together can reveal the mechanisms that drive a system.

The combined elements of this definition cover the effects (influences), the mechanisms (exchanges), and the experimentally tractable phase of interactions (Fig. 2A). While this definition offers a comprehensive and ontological perspective of fungal-bacterial interactions, it is important to recognize that multiple influence types, mechanisms of action, as well as any of the phases of interactions we proposed, can occur simultaneously at different scales of space and time (Fig. 2B and C). Unraveling these simultaneous pieces and placing them into the context in which they occur is central to a systems understanding of fungal-bacterial interactions.

## TOWARD CAUSES AND MECHANISMS IN FUNGAL-BACTERIAL INTERACTIONS

Fungal-bacterial interactions, as a developing field, must strive toward identifying causal relationships that are key to predicting system behavior, avoiding the path of correlation that has dominated modern microbiome science. To this end, we propose using Bradford Hill’s set of flexible guidelines (Hill’s criteria for causation [42]) to assess whether an association between a supposed cause and effect is merely a correlation, that is, a statistical relationship between variables with no clearly identified explanation, vs a true causal link with affirmation that one event is the result of the occurrence of another event(s) (43). Causation can be evaluated through multiple criteria, including strength of association (a strong statistical link between variables), temporality (the cause occurs before the effect), plausibility (a credible biological or mechanistic explanation exists), specificity (a cause leads to a particular effect), experiment (interventions or manipulations support the relationship), consistency (the association is observed across different studies and contexts), coherence (the relationship aligns with existing theoretical and empirical knowledge), biological gradient (the effect varies with the level of exposure), and analogy (similar causes are known to produce similar effects) (42, 43). The more of these criteria are fulfilled, the better we can attribute causation to an observed interaction.

Currently, network analysis has become a choice tool to make sense of complex microbiome data sets. It has paved the way for correlative assessment of fungal-bacterial interactions by identifying potential fungal-bacterial co-occurrence patterns (“interactions”), identifying keystone taxa, and correlating how environmental factors may influence network structural properties. While network properties resulting from hypothesis-driven experiments and supported by additional evidence can be powerful for unraveling causal relationships, when used in isolation, they lack the ability to identify the causality associated with broad microbial community assembly and interactions (44, 45). However, they can help identify mechanistic hypotheses to be tested and point toward previously unseen potential interactions (46). Once mechanistic hypotheses have been identified, they may be tested, and the components of the findings may be integrated to build a solid causal relationship by fulfilling Hill’s criteria for causation.

As causality implies that there will be an effect, the discovery of the mechanisms underlying the processes between cause and effect is crucial to understanding fungal-bacterial interactions as a system. In other words, we must also identify the connections and interactions among parts of the system (47). Bechtel and Abrahamsen (48) describe mechanisms as “a structure performing a function in virtue of its component parts, component operations, and their organization.” Let us examine this definition through the mechanisms of organic phosphorus mineralization and transport in soil (49). A microbial community composed of an arbuscular mycorrhizal fungus and a phosphate-solubilizing bacterium can form a “structure” that facilitates plant phosphorus uptake (the “function”). This structure arises from its component parts (fungal hyphae, bacterial cells, and their respective metabolic and physical traits, such as a hyphal network), their individual operations (soil exploration and phosphate solubilization), and their spatial and temporal organization (bacteria use the fungal hyphae to colonize soil compartments where they facilitate organic phosphorus mineralization, and the fungal network acts as a piping system that provides resources to the bacteria and transports phosphorus back to plant roots). This description of the mechanism is particularly powerful as it allows us to consider multiple factors (component parts) beyond the fungi and bacteria themselves, and whether these factors contribute directly (e.g., antibiosis, cell lysis) or indirectly (e.g., nutrient depletion, pH change) to their interactions (component operations) and outcomes (functions) (50). Critically, these components and component operations must be connected (organized) in a spatially and temporally explicit system (30).

In addition, it is important to recognize that mechanisms of fungal-bacterial interactions can be nested within higher-order combinations (“nestedness”), where the relationship between two species can modulate or be modulated by one or more external species. For example, the colonization of the fungus *Piriformospora indica* with its host plant can be enhanced by its association with *Rhizobium radiobacter* (51). Similarly, the pathogenicity of rice seedlings depends on the presence of various *Burkholderia*-related endobacteria within *Rhizopus microsporus* (52–54). In other cases, interactions between bacteria and competing wood-decay fungi can lead to unpredictable outcomes in the decomposition process (55). However, interactions involving multiple species with shifting roles present additional challenges for their categorization, as it becomes difficult to assign clear benefits or harms when participants in multispecies interactions cannot be confined to singular roles. These issues are amplified as the physical hyphal networks have the potential to connect multiple sets of organisms, each operating independently, with different metabolic abilities and in different microenvironments (Fig. 2). Such physical hyphal network connectedness is not only common but also necessary for the functioning of ecosystems as they allow the emergence of key properties like resilience, adaptation, stability, and homeostasis (56). We should therefore be aware that while mechanisms are a necessary vehicle of explanation in fungal-bacterial interaction systems, they are nevertheless limited due to the specificity, context-dependency, and the nestedness of the system. Despite these limitations, observing interactions in the light of mechanisms offer a rich perspective that bring us appreciably closer to understanding the nature of fungal-bacterial interactions.

## FUNGAL-BACTERIAL INTERACTIONS ACROSS TEMPORAL SCALES

An intrinsic property of interactions is that they unfold over time (57), exposing the underlying processes and mechanisms that cannot be captured with single snapshot experiments. Therefore, it is essential to understand fungal-bacterial interactions through time using longitudinal experiments. These experiments allow us to detect interactions that change with context, whether that context is intrinsic (e.g., phenology of the fungus or bacteria) or extrinsic (changing environmental/abiotic factors) to the system. For instance, bacterial communities inhabiting the fruiting bodies of fungi may shift from a mutualistic role to a pathogenic or parasitic role during the development of the fruiting body (58), or the reduction of an ectomycorrhizal fungal host can also reduce the relative abundance of fungal-associated *Burkholderia* (59). Time-resolved studies are also crucial for detecting time-delayed interactions, such as predator-prey cycles (e.g., mycophagy) (60), and for understanding how environmental disturbances, like drought, can lead to shifts toward alternative stable states in fungal-bacterial communities (61, 62). As both fungal and bacterial communities exhibit circadian oscillation, as well as seasonal variation (63–65), longitudinal experiments are essential for capturing these fluctuations and understanding their impact on the outcomes of the interactions. To this end, our proposed framework of the four phases of interactions (sense, response, feedback, and outcome; Fig. 2) can serve as a guide to designing longitudinal studies. By implementing treatments that modify the stable state of a system (i.e., any perturbations), we can transition from observing a single snapshot to revealing patterns, periodicity, irregularities, or alternative states. While concepts such as seasonality, predator-prey cycles, and shifts to alternative stable states are ideas gained from community ecology studies, they have yet to be appreciably explored in the context of fungal-bacterial interactions.

Considering that interactions are subject to dynamic processes that unfold over varying temporal scales, it is necessary to select appropriate longitudinal time scales in experimental designs that effectively build a mechanistic understanding of the system. However, the selection of the “right” time scale is not trivial because different ecological processes dominate at different temporal scales. Additional challenges in selecting time scales are that, in general, fungi and bacteria can have very different growth and turnover rates, with bacterial turnover rates being up to 10 times faster than fungi (65, 66). While both fungi and bacteria disperse effectively through the air, their arrival order on sterile substrates, such as those of newly opened flowers, leaves, roots, volcanic lava, or human-made substrates, can determine the outcomes of interactions (67, 68). This concept follows the well-established concept of “priority effects,” where the arrival order of one or more organisms can dictate the community development of the system (69). In laboratory inoculation experiments, this reality not only makes it challenging to perform the experiments but can also influence observations at earlier time points. For instance, in culture bioassays, bacteria can reach substantial biomass overnight, but it may take a couple of days before fungal hyphae reach large enough biomass to be effective in the interaction. Furthermore, bacteria tend to grow more robustly than fungi in liquid medium, so the choice of medium must also be considered. Inoculating these organisms simultaneously, whether into solid or liquid media, would inherently introduce priority effects into the bioassays. As a result, changes in the abundance and growth of bacteria and fungi, either inoculated simultaneously or sequentially, need to be evaluated within a narrow time window to allow the detection of small changes over brief periods (70), and interpretation must consider any priority effects introduced into the system. Conversely, longer time scales may be needed to observe certain types of interactions. For example, unknown selection pressures from the yeast *Debaryomyces hansenii* increased genomic and phenotypic diversification of the bacterium *Staphylococcus* after an extended co-culture period (71). Therefore, longitudinal experiments in fungal-bacterial interactions should be carefully designed to best reflect the most appropriate time scale of the system, including not only the variable generation times of fungi and bacteria but also their mutation rates. Established knowledge of the life cycles of the organisms, dynamics, and the processes associated with the phenomenon being investigated will help with this design. With an appropriately designed longitudinal study, we can transition from observing single snapshot events to resolving the mechanisms driving the phases of interactions.

## FUNGAL-BACTERIAL INTERACTIONS ACROSS SPATIAL SCALES

Morphological differences between fungi and bacteria (e.g., unicellular vs filamentous, cell dimensions, mobility, and colonization abilities) can have major effects on the spatial scale at which they interact, including the outcomes of measurements assessing interactions. Reconciling the differences among them is one of the biggest challenges in fungal-bacterial interaction studies. Smaller-size organisms like bacteria exhibit higher niche breadth and ease in searching for resources in microhabitats, but they have limited colonization abilities, particularly in heterogeneous environments that are not saturated with water (72). In contrast, fungi with their larger cell sizes have limited access to the smallest microenvironments, but their filamentous hyphal growth can actively explore different habitats and can spread over vast volumes (73). If we were to study these organisms independently, bacteria would operate at the micrometer to centimeter scale, whereas fungi would operate at the centimeter or even meter scale as they can develop extensive hyphal networks (74). However, in fungal-bacterial interactions, the spatial scale of interactions can change drastically. Hypothetically, immediate nutrient exchange between these two partners (whether externally or as endosymbionts) may operate at the micrometer to millimeter scale, while nutrient exchange across a network (e.g., a biofilm) may operate at the millimeter to centimeter scale, and motile bacteria dispersing on fungal hyphae have the potential to operate on the meter scale.

Adding to this already challenging spatial arena, antagonistic interactions can manifest as spatial patterns of coexistence and exclusion (75), creating distinct micro-communities that increase diversity and complexity over larger scales, such as an entire hyphal network (Fig. 2C). This rising complexity is particularly relevant in fungal-bacterial interactions where microenvironments can be connected by a network of hyphal bridges that facilitates exchange of nutrients, water, and energy among multiple bacterial subsystems, thereby expanding the spatial scale of interactions. Additionally, fungal-bacterial interactions can transcend spatial constraints through the release of volatile organic compounds (VOCs). For instance, VOCs produced by the fungal pathogen *Fusarium culmorum* can induce the production of secondary metabolites by the bacterium *Serratia plymuthica* (76), and multiple strains of actinomycetes can stimulate spore germination of the arbuscular mycorrhizal fungus *Gigaspora margarita* (77). This highlights the importance of selecting the appropriate spatial scale during experimental design, that is likely to determine which signals (or a flattening of signals) can be detected. For example, making observations at the micron scale to understand direct nutrient exchange across membranes is more appropriate than choosing the same scale to measure bacterial migration on fungal hyphae, since migration is likely to extend beyond the micron scale, affecting areas beyond the focal point of the study. Therefore, the selection of the right scale can fundamentally impact the experimental outcomes and interpretation of the results.

Since the mechanisms or mode of action of fungal-bacterial interactions change at both the spatial and temporal scales, selecting the most appropriate scale becomes a significant challenge. The microscale appears to be more sensitive to stochastic factors, leading to a collection of unpredictable and unrepeatable measurements (78). However, it is necessary to understand how the distribution and connectivity of components and component operations at the smaller scales may influence the behavior of larger-scale systems. To address these challenges, macroecology accepts loss of details at the smaller scale to gain predictability at higher scales (79, 80), employing higher-scale “black box” experiments to effectively answer questions that seem unsolvable when only focusing on the lowest scales (81). It is important to recognize that what is being measured in these black box experiments is an average of the processes across many microenvironments, possibly nested within each other at different scales. We argue that complex systems cannot be understood using any single scale due to emergent properties that arise from interactions among individual components of a system; they cannot be predicted nor explained by observing those components alone (82, 83). These emergent properties are common, if not defining characteristics of complex systems that can only be detected when comparing different scales (84). Therefore, we advocate for performing fungal-bacterial interaction experiments at different scales, followed by synthesizing the results to build a more comprehensive understanding of the system. For example, to understand how fungal-bacterial interactions can reduce nitrification in soil, nutrient exchange and transcriptional changes may be measured at the smaller scale (e.g., single cell or on a single aggregate of soil), connected to the outcomes within individual soil samples (e.g., 10 g), and combined as outcomes of the processes at the landscape scale (e.g., a field) (85, 86). A primary goal of a cross-scale understanding of a system is to eventually be able to translate how complex fungal-bacterial interactions at the smallest scales drive ecosystem function, an area that remains amongst the biggest challenges in microbial ecology.

## EMBRACING THE COMPLEXITY

To comprehensively understand fungal-bacterial interaction systems and connect interactions to ecosystem function, we must engage with the inherent complexity and systematically connect the underlying operations. As such, we must develop approaches that can reliably predict the relationship between interactions and ecosystem function. However, creating generalizable mechanistic models across the broad diversity of interacting organisms, environments, and temporal dynamics is inherently difficult (7). The difficulty in determining the most relevant scale for a system, combined with logistical challenges, has so far prevented studies from accessing a broader scale spectrum (87). Consequently, methods such as mechanistic modeling are often confined to a single scale or experimental setup and thus are limited in their ability to grapple with the complexity of natural systems (88). This makes higher-scale approaches, such as those used in macroecology and systems ecology, appealing as they allow the detection of consistent patterns across contexts without necessarily resolving the underlying mechanisms (79, 80). In general, mechanistic models can clarify paradoxical processes in complex systems (89, 90), while macroecological approaches assess whether laboratory-identified mechanisms hold ecological relevance (91). Critically, both are needed to construct “theories of averages,” a framework that reveals regularities (i.e., recurring, predictable patterns) at specific levels of organization. These regularities emerge because as the scale of observation increases, the influence of stochastic micro-scale events diminishes, giving rise to more predictable patterns, such as mean community composition, stable interaction outcomes, or aggregate ecosystem functions (7, 92). Following these concepts, we may hypothesize that fungal-bacterial interactions could stabilize community composition, including their networks of interactions, and provide aggregate ecosystem functions, such as nutrient mineralization or carbon sequestration.

We acknowledge that there is no single “right” scale at which to study fungal-bacterial interactions and advocate for research strategies that integrate mechanistic details with a systems-level perspective. This requires a pluralistic perspective, where diverse models are viewed as complementary lenses on a shared underlying reality. Critically, the focus should shift from continually parameterizing the accuracy of the model to understanding how the observed changes are intertwined with complex system characteristics. In practice, this entails explicitly defining the spatial and temporal scales most relevant to the system under study, as well as the number and nature of interacting biotic and environmental components. This includes identifying spatial compartments (e.g., habitat patches), temporal phases (e.g., seasonality, circadian oscillations), and adjacent systems whose dynamics may influence or be influenced by the focal interaction network (Fig. 2). Making these boundaries and connections explicit can help prevent treating interactions as independent events while revealing hidden effects that ripple across scales. Coordinating this diversity of scales and contexts enables disparate research approaches to inform and reinforce one another, fostering a more comprehensive understanding of fungal-bacterial interactions.

**Fig 3:**
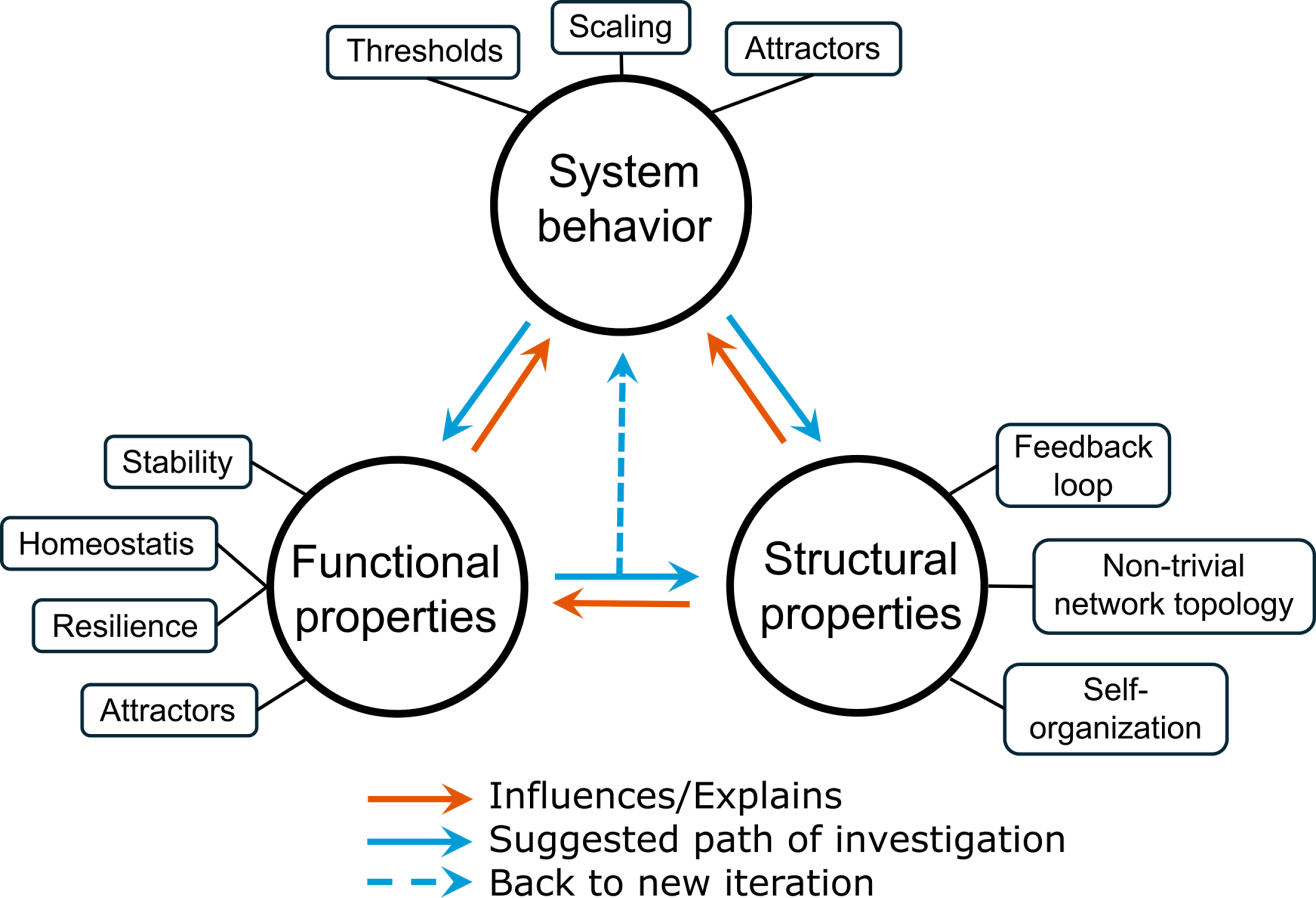
Iterative approach to assess complexity. To elucidate the mechanisms driving the complexity of fungal-bacterial interactions, we propose an iterative, scale-based approach. The process begins by testing system behavior across scales to detect when new properties arise. The state of the system at turning points is then examined to determine how structural and functional attributes change and influence the properties of interest. The figure illustrates an idealized workflow for each iteration: first, assess how the model responds to a change in scale, then evaluate associated structural and functional shifts, and finally, decide whether additional iterations (i.e., further scale changes) are needed. Iterations may also explore different types of scales (spatial, temporal, or taxonomic).

To operationalize this pluralistic framework, we propose a three-step iterative approach that can be applied across all scales and move in any direction (Fig. 3). For instance, an experiment focusing on a single time point might be developed toward a longitudinal study where the length of the experiment and the time between analyses are increased at each iteration. It would be just as appropriate to start with a macroecology study and reiterate the experiment and sampling, lowering the distance or size of the sample at each iteration. After identifying the starting place, the first step is to experimentally test the behavior of the system and determine whether the properties or patterns change across spatial, temporal, or taxonomic scale (a phenomenon known as scaling). Determining how changes happen can help detect the existence of thresholds (points at which a small change in system conditions produces a disproportionately large change in the system itself) and attractors (a state toward which the system properties tend to converge). After characterizing the behavior of the system across scales, attention can turn to how functional and structural properties shift around critical thresholds or inflection points. Functional properties (emergent properties that confer specific system-level capacities) are particularly important to consider, as they often represent desirable features, such as resilience, homeostasis, or stability. Conversely, chaotic behavior, characterized by small differences in initial conditions leading to large, deterministic divergences in system outcomes, represents an undesired dynamic that can also emerge or disappear as scale changes. And finally, investigating the structural characteristics of complex systems, such as non-trivial network topology, self-organization, or feedback loops, can reveal mechanisms that drive the emergent properties of the system.

The roadmap we drew here is far from complete and will require iterative adjustments, or perhaps introduce radical new approaches to understand complexity. It currently does not cover each scale independently, nor does it cover all the characteristics of a complex system. It outlines a pragmatic form of reductionism, not to simplify fungal-bacterial interactions to its minimal components, but to isolate and study the key drivers of their complexity (Fig. 3), ultimately supporting the development of integrative, system-level models through iterative synthesis. Yet, this approach may not be enough. A contrasting newer approach that diverges radically from traditional thinking into complex systems is to employ artificial intelligence implemented through machine learning models to build connections between component parts and component operations (93). In these approaches, models are often trained on unconstrained data sets without the need for individual components and identification of scale. Ideally, the trained models would be able to recognize deep and complex patterns, including interactions, within the system (94, 95). However, a tremendously large amount of high-quality data would be necessary to train accurate models (e.g., deep learning) that can build predictive, and even causal connections among the parts of a system (96). While this area of research has potential to help us tease apart the complexity of systems that have eluded traditional approaches, deep thinking and inquiry into scientific questions must remain at the forefront of any approaches used to understand fungal-bacterial interactions. By adopting integrated approaches, grounded in systems thinking but open to reductionist inquiry, as well as newer approaches, we may be better equipped to tackle one of the central challenges in this subfield of microbial ecology: how fungal-bacterial interactions at the microscopic scale give rise to macroscopic patterns and functions in ecosystems.

## CONCLUDING REMARKS

As we navigate the complexity of fungal-bacterial interactions across space and time, amalgamated through conceptual or ecosystem models, the studies we conduct should support the mechanistic and causal understanding of our system. When designing our experiments, we must consider the different scales of space and time, wrapped within nested complexity. Ideally, data generated through qualitative, quantitative, and hypothesis-driven experiments would support the sense, response, feedback, and outcome phases of interactions. While current ecological theory may provide a grounded framework, the nature of interactions lies in emergent properties, which require our constant awareness of discoveries. It took a surprisingly long time for bacteriology and mycology to build bridges, but we envision that cooperation across the diverse perspectives and curiosities surrounding fungal-bacterial interactions would be as swift as it is indispensable. We cannot perform this task alone. Although efforts are being made to centralize knowledge gleaned from fungal-bacterial interaction research (89, 90), explicit community coordination is necessary. As researchers, we must, ourselves, build a network of scientists, organized in multiple subgroups, interacting with each other through multiple feedback loops and stochastic encounters. Together, we can build a comprehensive understanding of fungal-bacterial interactions within and across systems. As fungal-bacterial interactions are becoming a topic of interest at the forefront of microbial ecology, a coordinated community of fungal-bacterial interaction researchers will be able to develop actionable knowledge, build an integrated understanding of the interaction system, and uncover emergent unknowns.
